# Antithrombotic therapy for a case report of acute myocardial infarction after laparoscopic radical cystectomy

**DOI:** 10.3389/fphar.2024.1477715

**Published:** 2025-01-03

**Authors:** Zilong Wang, Huisheng Yuan, Junhao Chu, Shishuai Duan, Zhihui Zhang, Changze Song, Muwen Wang

**Affiliations:** ^1^ Department of Andrology, The Seventh Affiliated Hospital, Sun Yat-Sen University, Shenzhen, China; ^2^ Department of Urology, Shandong Provincial Hospital, Cheeloo College of Medicine, Shandong University, Jinan, China; ^3^ Department of Urology, Shandong Provincial Hospital Affiliated to Shandong First Medical University, Jinan, China; ^4^ Scientific Research Center, The Seventh Affiliated Hospital, Sun Yat-Sen University, Shenzhen, China

**Keywords:** antithrombotic therapy, acute myocardial infarction, radical cystectomy, dual antiplatelet therapy, percutaneous coronary intervention, coronary angiography, urothelial carcinomas of the bladder, case report

## Abstract

**Background:**

Radical cystectomy constitutes the standard therapeutic approach for high-risk urothelial carcinomas of the bladder. Contemporary guidelines advise urologists to discontinue anticoagulation therapy during the perioperative period to mitigate the risk of significant intraoperative or postoperative hemorrhage. Nevertheless, in elderly patients with a history of coronary artery disease, the cessation of anticoagulant medication elevates the risk of acute myocardial infarction, thereby posing a substantial threat to their survival. Therefore, the necessity and optimal strategy for anticoagulation therapy in patients with acute myocardial infarction following radical cystectomy remains a subject of ongoing debate. This study aims to contribute clinical insights for clinicians to manage high-risk patients with acute myocardial infarction post-major surgery.

**Methods and results:**

The 64-year-old male patient was admitted for multiple high-grade urothelial carcinomas of the bladder. The preoperative computed tomography angiography revealed intra-luminal stenosis of the coronary arteries. However, the patient declined further assessment via preoperative coronary angiography, thereby precluding the accurate prediction of postoperative myocardial infarction risk. The patient subsequently underwent laparoscopic radical cystectomy with Bricker conduit urinary diversion and the postoperative pathological examination confirmed the diagnosis of high-grade urothelial carcinoma (T1N0M0, G3). Regrettably, on the first postoperative day, the patient experienced an acute anterior wall ST-segment elevation myocardial infarction. Consequently, the patient underwent emergency percutaneous coronary intervention and was administered dual antiplatelet therapy consisting of aspirin and ticagrelor. The daily pelvic fluid drainage, routine blood and coagulation parameters remained within normal ranges. Following the second percutaneous coronary intervention and dual antiplatelet therapy, the patient was discharged after 2 days. Over a 3-year follow-up period, all hematological parameters consistently remained within normal ranges, and there were no incidents of bleeding or anastomotic leakage.

**Conclusion:**

This study demonstrates that postoperative percutaneous coronary intervention, in conjunction with continued dual antiplatelet therapy, is a safe and effective antithrombotic strategy for managing perioperative acute myocardial infarction. This finding suggests a potential paradigm shift in the management of antithrombotic therapy for high-risk surgical patients, advocating for a tailored approach rather than the routine discontinuation of such therapy.

## 1 Introduction

Urothelial carcinomas of the bladder are among the fourth most prevalent malignancies among male worldwide, accounting for an estimated 6% of newly diagnosed cancers and 4% of cancer-related mortality (Siegel et al., 2024). Despite the fact that approximately 70%–75% of bladder cancers manifest as non-muscle-invasive bladder cancer (NMIBC) (Lopez-Beltran et al., 2024), radical cystectomy constitutes the standard therapeutic approach for multiple, high-grade and/or large-sized NMIBC (Sim et al., 2019; Babjuk et al., 2022). Regardless of the standardization of surgical procedures for radical cystectomy and other major surgeries, patients with pre-existing cardiovascular and cerebrovascular conditions remain at considerable risk for severe complications, including acute myocardial infarction (Babjuk et al., 2022; Cathomas et al., 2022). Thus, determining effective treatment and prevention strategies for antithrombotic therapy in these patients is critically important.

Primary percutaneous coronary intervention (PCI) remains the gold standard in patients with ST-elevation myocardial infarction (STEMI) by re-establishing blood flow to the occluded myocardium through techniques (He et al., 2022), including balloon angioplasty and stent implantation (Jeger et al., 2020). The introduction of drug-eluting stents, which release pharmacological agents like sirolimus and paclitaxel via polymer coatings on bare metal stents, has further augmented the efficacy of PCI by mitigating in-stent restenosis and decreasing the necessity for repeat revascularization procedures (Fu et al., 2020; Suna et al., 2018; Giustino et al., 2017). However, postoperative dual antiplatelet therapy (DAPT), consisting of oral aspirin and P2Y12 inhibitor, is recommended post-PCI to effectively reduce the risk of cardiovascular ischemic events (Valgimigli et al., 2024). But this implement concurrently increases the likelihood of bleeding complications (Orme et al., 2018). The implementation of drug-coated balloons has been shown to potentially reduce the incidence of late stent thrombosis (Secemsky et al., 2019), underscoring the critical necessity for a balanced approach in antithrombotic therapy to effectively prevent both thrombotic and hemorrhagic events.

Contemporary guidelines advise urologists to discontinue anticoagulation therapy during the perioperative period to mitigate the risk of significant intraoperative or postoperative hemorrhage (Albisinni et al., 2022). However, this strategy may increase the likelihood of intraoperative or postoperative thrombosis, especially in patients with malignancies who also have concomitant cardiovascular or cerebrovascular conditions (Friedrich et al., 2019). Furthermore, patients with malignancies frequently exhibit a hypercoagulable state (Debergh et al., 2010). Researchers indicate that maintaining antithrombotic therapy does not significantly elevate intraoperative blood loss. And it cannot increase perioperative and postoperative transfusion and complication rates (Furrer et al., 2022; Wessels et al., 2019). Consequently, it is crucial to meticulously select an antithrombotic therapy strategy tailored to patients with malignancies during the perioperative period.

The rate of perioperative acute myocardial infarction (AMI) in urological surgeries is 0.36% (Jiang D. D. et al., 2021) and the in-hospital mortality rates of this condition have reached up to 15.2% (Devereaux et al., 2011). Therefore, it is crucial to receive treatment immediately in case of a perioperative thrombotic event. The application of PCI in conjunction with continued DAPT is fraught with challenges largely due to the risk of possible postoperative hemorrhage.

In this study, we described a 64-year-old male patient with multiple high-grade urothelial carcinomas of the bladder who experienced an acute STEMI on the first postoperative day subsequent to laparoscopic radical cystectomy. The patient was administered antithrombotic therapy, which included PCI and dual antiplatelet therapy with aspirin and ticagrelor. Throughout the treatment, the patient maintained stable routine blood and coagulation indices, with no occurrence of hemorrhage or anastomotic leakage. The objective of this study was to furnish clinicians with critical insights for managing similar patients with acute myocardial infarction during the perioperative period.

## 2 Methods and results

### 2.1 Preoperative and operative conditions

**FIGURE 1:**
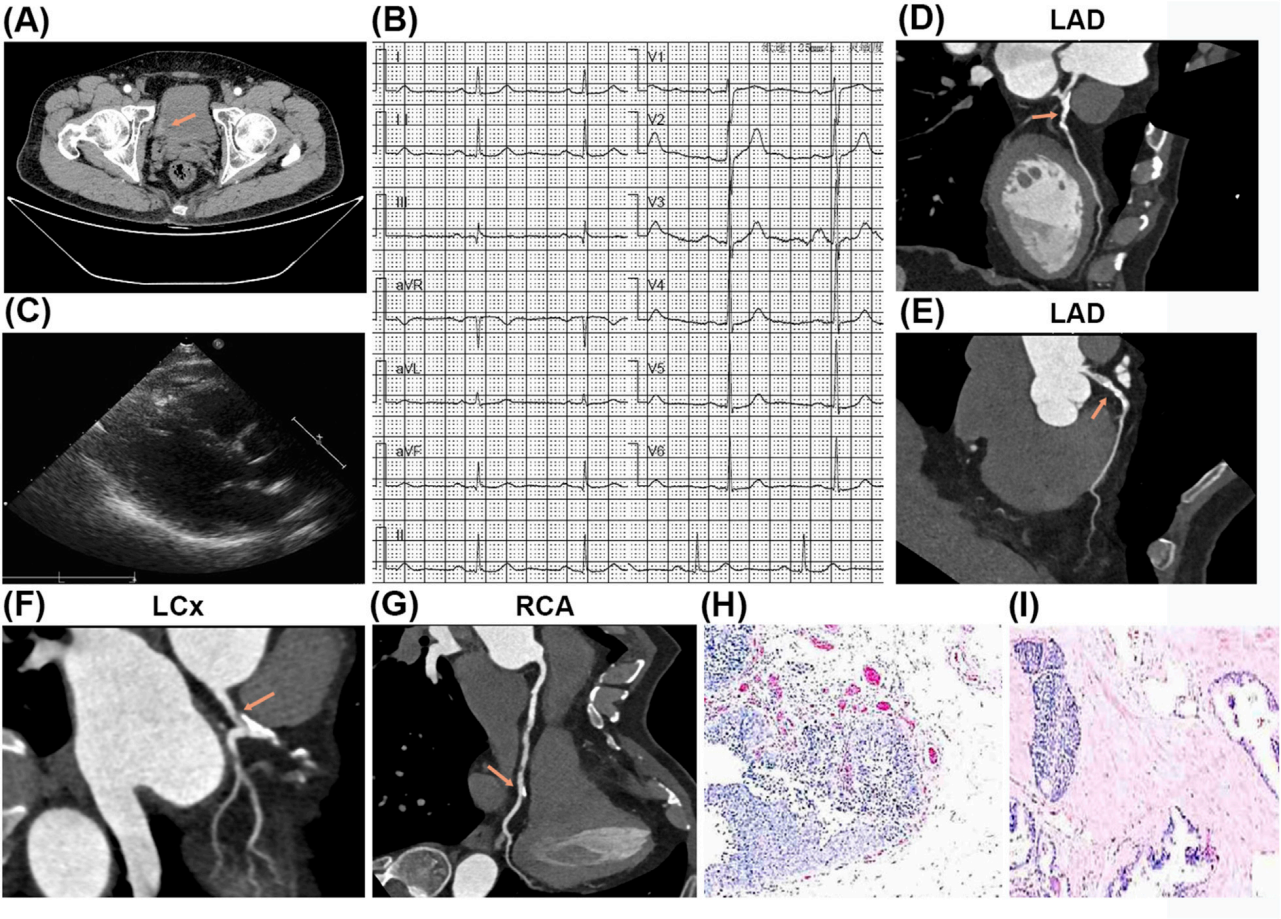
The results of preoperative testing and postoperative pathology examination. **(A)** The preoperative computed tomography (CT) revealed multiple papillary lesions located on the right lateral bladder wall, bladder dome, and bladder neck, with the largest lesion measuring 2.0 × 1.5 cm. **(B)** The preoperative electrocardiograms (ECG) showed ST-segment slightly depression (V4-V6). **(C)** The preoperative cardiac ultrasound indicated no abnormality. **(D–G)** The computed tomography angiography (CTA) revealed luminal stenosis exceeding 50% in the left main coronary artery (LM), left anterior descending artery (LAD), and left circumflex artery (LCx) **(D–F)**, as well as approximately 30% stenosis in the distal segments of the right coronary artery (RCA) **(G)**. **(H, I)** The postoperative pathological examination showed high-grade urothelial carcinomas of the bladder.

The 64-year-old male patient was admitted to the urology department for multiple high-grade urothelial carcinomas of the bladder with severe gross hematuria on 15 September 2021. He had no prior history of diabetes mellitus, chronic cerebrovascular disorders, psychological conditions, genetic disorders, or malignant neoplasms, and there was no familial history of these conditions. Preoperative computed tomography (CT) revealed multiple papillary lesions located on the right lateral bladder wall, bladder dome, and bladder neck, with the largest lesion measuring 2.0 × 1.5 cm (Figure 1A). Preoperative electrocardiograms (ECG) indicated mild ST-segment depression in leads V4-V6 (Figure 1B), while cardiac ultrasound demonstrated a normal left ventricular ejection fraction (LVEF) of 61% (Figure 1C). Computed tomography angiography (CTA) revealed luminal stenosis exceeding 50% in the left main coronary artery (LM), left anterior descending artery (LAD), and left circumflex artery (LCx), as well as approximately 30% stenosis in the distal segments of the right coronary artery (RCA) (Figures 1D–G). Nevertheless, the patient opted against undergoing a preoperative coronary angiography (CAG) examination.

Following a multidisciplinary team (MDT) discussion encompassing urology, cardiology, anesthesiology, and intensive care specialists, the patient underwent a laparoscopic radical cystectomy with Bricker conduit urinary diversion on 17 September 2021. The surgical procedure was executed without complications through maintaining stable intraoperative vital signs and minimal blood loss (100 mL). Postoperative pathological analysis confirmed the presence of high-grade urothelial carcinomas measuring 4 × 0.5 cm, with invasion into the lamina propria (T1N0M0, G3) (Figures 1H, I).

Postoperatively, continuous ECG monitoring indicated no abnormalities, and routine blood tests were within normal reference ranges, including hemoglobin (Hb) at 156 g/L (normal reference range 130–175 g/L), platelet count (PLT) at 167×10^9^/L (normal reference range 125–350 × 10^9^/L), red blood cell count (RBC) at 5.45 × 10^12^/L (normal reference range 4.30–5.80 × 10^12^/L), and hematocrit (HCT) at 50.2% (normal reference range 40.0%–50.9%). Coagulation assays demonstrated a D-dimer level of 1.17 mg/L (normal reference range 0–0.55 mg/L), a prothrombin time (PT) of 13.70 s (normal reference range 10.7–14 s), an activated partial thromboplastin time (APTT) of 32.90 s (normal reference range 23–37 s), and a prothrombin time-international normalized ratio (PT-INR) of 1.05 (normal reference range 0.8–1.2). Myocardial enzyme levels were marginally elevated, with myoglobin at 98.20 ng/mL (normal reference range 28–72 ng/mL), hypersensitive troponin (HS-TnT) at 9.03 pg/mL (normal reference range 0–14 pg/mL), serum creatine kinase-MB (CK-MB) at 2.78 ng/mL (normal reference range 0.10–4.94 ng/mL), and N-terminal pro-B-type natriuretic peptide (NT-ProBNP) at 235 pg/mL (normal reference range 0–125 pg/mL). Daily pelvic fluid drainage was measured at 50 mL.

### 2.2 Antithrombotic therapy for postoperative myocardial infarction

**FIGURE 2:**
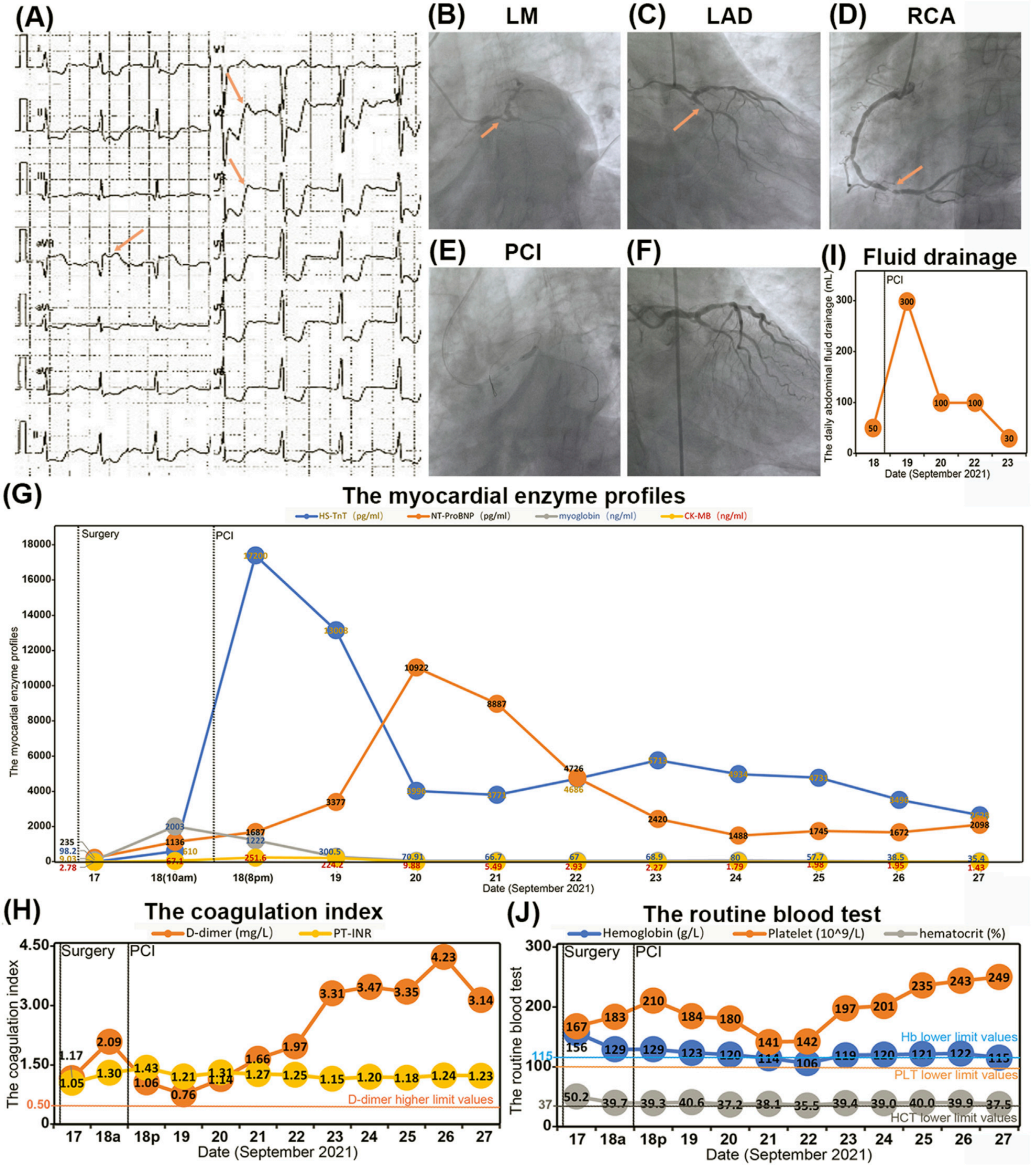
The conditions of acute myocardial infarction and antithrombotic therapy. **(A)** The ECG showed ST-segment depression in aVR and ST-T-segment depression in V2-V6. **(B–D)** The CAG identified significant stenosis ranging from 70% to 90% in the LM, LAD, and LCx arteries, with approximately 90% stenosis observed in the distal RCA. **(E)** The first percutaneous coronary intervention (PCI) procedures. **(F)** The CAG showed the luminal stenosis from LM to LAD have been lifted after PCI. **(G–J)** The blood index during the process of antithrombotic therapy including myocardial enzyme profile **(G)**, coagulation index **(H)**, pelvic drainage **(I)** and routine blood test **(J)**.

Fourteen hours postoperatively, on 18 September 2021 (postoperative day 1), the patient exhibited signs of delirium, accompanied by hypotension with a blood pressure reading of 86/46 mmHg (normal reference range 90/60–140/90 mmHg). ECG findings demonstrated ST-segment depression in lead aVR and ST-T segment depression across leads V2 to V6 (Figure 2A). Laboratory results indicated elevated myocardial enzyme levels and coagulation indices, with myoglobin at 2003.00 ng/mL, HS-TnT at 610.0 pg/mL (normal reference range 0–14 pg/mL), CK-MB at 67.10 ng/mL (normal reference range 0.10–4.94 ng/mL), NT-ProBNP at 1,136 pg/mL (normal reference range 0–125 pg/mL), and D-dimer at 2.09 mg/L (normal reference range 0–0.55 mg/L). Bedside cardiac ultrasound identified a myocardial infarction localized to the anterior wall of the left ventricle, with a LVEF of 52% (normal reference range 50%–70%). The diagnosis was acute anterior wall STEMI complicated by cardiogenic shock.

Given the recent major surgical intervention, the treatment strategy necessitated meticulous balancing of the bleeding risk against the imperative for prompt revascularization. The therapeutic regimen included a subcutaneous injection of 40 mg low-molecular-weight heparin (LMWH) and dual antiplatelet therapy (DAPT) comprising 30 mg oral aspirin and 180 mg ticagrelor. Furthermore, 20 mg norepinephrine was administered intravenously to sustain adequate blood pressure. Emergency coronary angiography (CAG) identified significant stenosis ranging from 70% to 90% in the LM, LAD, and LCx arteries, with approximately 90% stenosis observed in the distal RCA (Figures 2B–D). The patient subsequently underwent percutaneous coronary intervention (PCI), which included drug-coated balloon angioplasty and the deployment of a 3.0 × 23 mm drug-eluting stent extending from the LM to the LAD (Figure 2E). Post-procedural CAG confirmed the successful resolution of the stenosis, achieving a Thrombolysis in Myocardial Infarction (TIMI) flow grade of 3 (Figure 2F). Following PCI, the patient’s myoglobin levels decreased to 1,222.00 ng/mL (normal reference range 28–72 ng/mL) (Figure 2G), and D-dimer levels reduced to 1.06 mg/L (normal reference range 0–0.55 mg/L) (Figure 2H). However, an increase in HS-TnT to 17,200.00 pg/mL (normal reference range 0–14 pg/mL) and a stable level of NT-ProBNP at 1,687 pg/mL (normal reference range 0–125 pg/mL) (Figure 2G) necessitated the intravenous administration of 5 mg tirofiban to enhance antithrombotic efficacy.

Fortunately, the daily pelvic fluid drainage was limited to 300 mL (Figure 2I), and all routine blood indices remained within normal ranges (Figure 2J). The combination of antithrombotic therapy and PCI did not result in active bleeding at the surgical site on postoperative day 1 (POD1). The patient’s DAPT regimen was maintained as 30 mg of oral aspirin and 180 mg of ticagrelor for approximately 1 month.

### 2.3 Postoperative conditions after AMI and antithrombotic therapy

**FIGURE 3:**
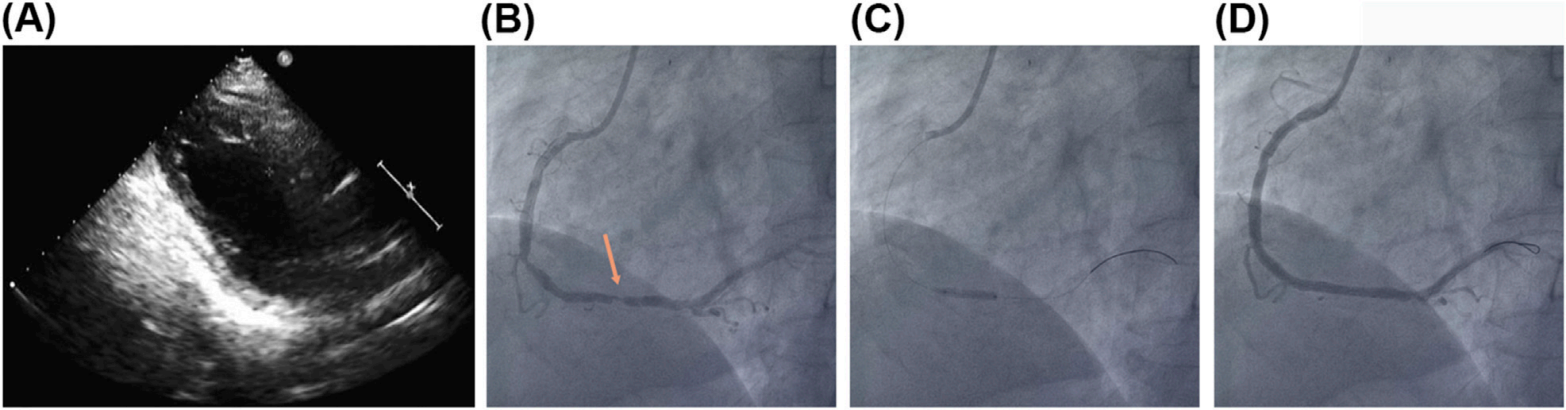
The second PCI therapy. **(A)** The cardiac ultrasound indicated segmental left ventricular wall motion abnormalities. **(B)** The CAG showed luminal stenosis at the distal segments of RCA of about 90%. **(C)** The second PCI procedures. **(D)** The CAG confirmed the luminal stenosis at RCA have been lifted.

Commencing on 19 September 2021 (postoperative day 2), there was a consistent declination in myocardial enzyme levels, including myoglobin, HS-TnT, and CK-MB (Figure 2G). Cardiac ultrasound performed on 22 September 2021 (postoperative day 5), indicated segmental abnormalities in left ventricular wall motion, with a LVEF of 60% (Figure 3A). The daily volume of pelvic fluid drainage remained below 100 mL (Figure 2I), and routine blood indices remained stable over the subsequent 4 days (Figure 2J). These observations suggest that the combination of aspirin, ticagrelor, tirofiban, and PCI therapy did not induce active bleeding at the surgical site. Therefore, the pelvic drain was removed on 23 September 2021 (postoperative day 6) as the daily pelvic fluid drainage had decreased to 30 mL (Figure 2I). Additionally, routine blood indices remained within normal ranges throughout the follow-up period (Figure 2J).

On 27 September 2021 (postoperative day 10), a reexamination of myocardial enzyme levels indicated reductions in myoglobin, HS-TnT, CK-MB, and NT-ProBNP to 35.40 ng/mL, 2,628.0 pg/mL, 1.43 ng/mL, and 2098 pg/mL, respectively (Figure 2G). However, an increase in D-dimer levels to 4.23 mg/L (Figure 2H) prompted a CAG on postoperative day 11. The CAG confirmed patency from the LM to the LAD artery but revealed significant stenosis (50%–70%) in the proximal and middle segments of the LCx and approximately 90% stenosis in the distal RCA (Figure 3B). Consequently, an additional PCI was conducted on 28 September 2021 (postoperative day 11), which included percutaneous coronary balloon angioplasty and the deployment of two drug-eluting stents (3.0 × 23 mm and 3.5 × 23 mm) in the RCA (Figure 3C). Intraoperative CAG confirmed the resolution of stenosis in the RCA, achieving Thrombolysis in TIMI flow grade 3 (Figure 3D). Post-PCI, a total of 12.5 mg of tirofiban was administered intravenously.

Throughout the hospitalization period following both PCI procedures and antithrombotic therapy, routine blood and coagulation indices remained stable and within normal ranges, negating the need for blood transfusion treatment (Figures 2H, J). The patient was subsequently discharged on 30 September 2021 (postoperative day 13).

### 2.4 Postoperative follow-up conditions

**FIGURE 4:**
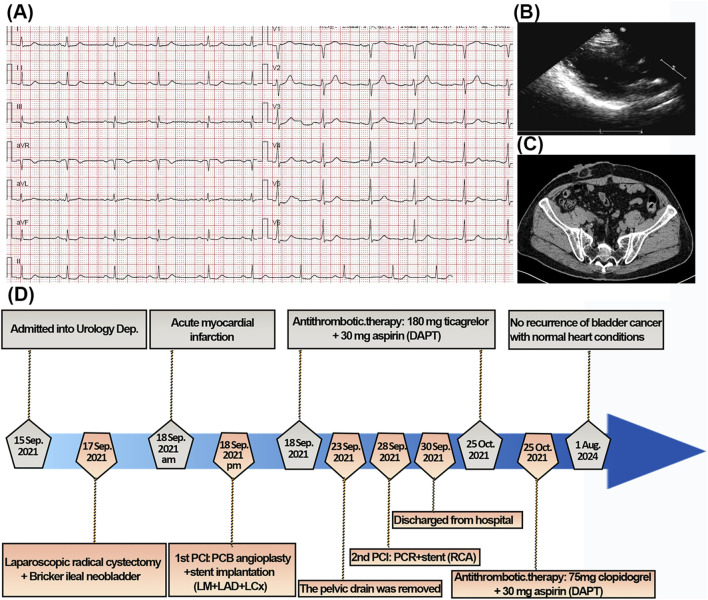
The 3-year follow-up conditions. **(A)** The latest ECG showed ST-segment depression in V2-V6. **(B)** The cardiac ultrasound indicated myocardial infarction of left ventricle. **(C)** The abdominal CT showed no recurrence and metastasis of bladder cancer and excellent ileal bladder substitute. **(D)** The flowchart of the timeline for the diagnosis and treatment schedule.

One-month post-discharge, the patient’s DAPT regimen was modified to 30 mg of oral aspirin and 75 mg of clopidogrel, owing to the heightened risk of major hemorrhage associated with prolonged ticagrelor use (Turgeon et al., 2020). Throughout a 3-year follow-up period, the patient expressed satisfaction with the treatment outcomes. The latest evaluations revealed ST-segment depression in leads V2-V6 (Figure 4A) and a LVEF of 55% (Figure 4B).

The latest abdominal CT showed no recurrence and metastasis of the urothelial carcinomas of the bladder (Figure 4C). Additionally, the anastomosis sites exhibited excellent healing, with no indications of hemorrhage or anastomotic leakage. The patient’s DAPT regimen was maintained as 30 mg of oral aspirin and 75 mg of clopidogrel for approximately 1 year and then modified to 30 mg of oral aspirin up to now. The diagnostic and treatment timeline are comprehensively summarized in Figure 4D.

## 3 Discussion

This case report underscores a significant challenge in the management of patients with high-risk urothelial carcinomas of the bladder who also present with a history of coronary artery disease. The dilemma revolves around the perioperative management of antithrombotic therapy. Discontinuation of this therapy may mitigate the risk of intraoperative and postoperative hemorrhage. However, it concomitantly elevates the risk of potentially fatal myocardial infarctions. The case illustrates that the implementation of rigorous and standardized laparoscopic radical cystectomy with Bricker conduit urinary diversion, followed by postoperative PCI in conjunction with continued DAPT using ticagrelor and aspirin, can be both safe and effective. Additionally, personalized management of anticoagulant therapy is also essential for patients undergoing major surgical procedures. In situations where the risk of myocardial infarction is heightened, the careful continuation of dual antiplatelet therapy can be warranted, contingent upon stringent adherence to surgical and monitoring protocols. This finding suggests a potential paradigm shift in the management of antithrombotic therapy for high-risk surgical patients, advocating for a tailored approach rather than the routine discontinuation of such therapy.

Patients undergoing surgery for malignancies frequently exhibit a hypercoagulable state, which heightens their susceptibility to thrombus formation, luminal stenosis, and AMI during the perioperative period (Friedrich et al., 2019; Debergh et al., 2010). Research indicates that the incidence of perioperative AMI in urological surgeries is 0.36%, with radical cystectomy representing one of the highest risks for AMI complications (Jiang Y. et al., 2021). Moreover, three-vessel coronary artery disease has been identified as a significant predictor of AMI, particularly in elderly patients over 60 years old (Han et al., 2022; Janardhanan et al., 2006). In this instance, a 64-year-old patient was identified with significant luminal stenosis in the LM, LAD, LCx, and RCA through preoperative coronary CTA. Based on these findings, it is recommended that elderly patients with established coronary artery disease undergo coronary CTA before major surgical interventions to identify potential contraindications to minimally invasive procedures.

However, although sensitivity and specificity of coronary CTA have improved to 93% and 92%, respectively, the accuracy of coronary CTA in evaluating the severity of a luminal stenosis is still lower than coronary angiography (CAG) (Nurmohamed et al., 2024; Forrest et al., 2023). Therefore, in cases where severe stenosis is detected, CAG should be considered to inform decisions regarding perioperative antithrombotic therapy. What is more severe is that the in-hospital mortality rates of perioperative AMI have reached up to 15.2% (Devereaux et al., 2011). Therefore, the effective interventions are of vital importance. In a propensity-matched cohort of 34,650 patients with perioperative AMI, an invasive approach was associated with lower in-hospital mortality than a conservative approach (8.9% vs. 18.1%; *p* < 0.001), but it was also associated with increased rates of postoperative hemorrhage (Smilowitz et al., 2017). More recently, a Danish cohort found that 38.5% of patients with perioperative AMI underwent CAG (Korsgaard et al., 2022). And in another study of patients diagnosed with perioperative AMI, only 21% underwent CAG in which 37% receiving some form of revascularization like PCI (Smilowitz et al., 2017). Therefore, a case can be made for the adoption of a more aggressive and invasive management approach in patients with perioperative AMI with high-risk features.

Percutaneous coronary intervention (PCI) with stent implantation has become the most widely used treatment for coronary artery disease (Suna et al., 2018). For patients with non-cardiac surgery after preoperative PCI, previous studies have demonstrated that the rates of myocardial infarction and cardiac death occurring in these patients were 0.65%–8.09% and 0.22%–0.85%, respectively (Kang et al., 2024; Wang et al., 2024; Graham et al., 2018). These data were well below the in-hospital mortality rates of perioperative AMI. Meanwhile, the rates of major bleeding were only 1.33%–5.83% (Kang et al., 2024; Wang et al., 2024; Graham et al., 2018). In summary, we recommended that PCI and antiplatelet therapy should be considered to patients with high-risk cardiovascular diseases before limited-stage or elective non-cardiac surgery. Active surveillance or conservative management can be performed to monitor the progression of primary diseases during the period of DAPT. Discontinuation of antiplatelet therapy has the potential to increase the risk of major perioperative adverse cardiovascular events, with stent thrombosis being the most feared because of its high associated morbidity and mortality (Wang et al., 2024). The discontinuation of aspirin and other antiplatelet agents should be reserved for patients with high bleeding risk and a comparably low ischemic risk, with procedures performed in PCI-capable hospitals so patients can immediately be treated in case of a perioperative thrombotic event (Halvorsen et al., 2022). And antiplatelet therapy was recommended to be resumed in all these patients no later than 48 h after non-cardiac surgery, unless contraindicated (Angiolillo, 2024). For patients who are at a moderate-to-high risk of cardiovascular events, the continuation of antiplatelet therapy is recommended during the perioperative period (Wang et al., 2024; Douketis et al., 2012).

Atherosclerosis in its initial stages is marked by the presence of non-calcified plaques (NCP) and mixed plaques comprising extracellular lipids and fibrous tissue. NCPs are more commonly observed in male smokers and exhibit a higher propensity for rupture and thrombosis, which can precipitate acute coronary syndrome (Al-Muhaidb et al., 2021). Researchers have demonstrated that non-calcified obstructive coronary artery plaques, which are independently positive associated with the duration of smoking, are identified as an independent risk factor for adverse cardiovascular events, including AM), within a 2-year period (Jiang D. D. et al., 2021; Miller et al., 2023). This patient had a prolonged history of smoking, and the preoperative coronary CTA revealed the presence of NCP in the LCx and RCA, thereby categorizing him as high-risk for perioperative AMI. Coronary CTA proves to be a valuable tool for detecting such plaques and assessing the severity of luminal stenosis, which facilitates improved perioperative risk management (Meah et al., 2022).

Previous research has demonstrated that the continuation of antiplatelet therapy with aspirin during the perioperative period of non-cardiac surgery does not significantly elevate the risk of intraoperative blood loss, transfusion rates, or perioperative complications (Wessels et al., 2019; Furrer et al., 2022; Albisinni et al., 2022). However, there is a paucity of data regarding the continuous use of DAPT with aspirin in combination with ticagrelor or clopidogrel during this period. In this instance, the patient underwent PCI and was administered DAPT comprising aspirin and ticagrelor within 24 h postoperatively. Despite this intervention, routine blood tests and pelvic drainage metrics remained stable within normal limits throughout the hospitalization period, and no blood transfusion was necessitated. These observations suggest that DAPT can be safely managed in similar clinical scenarios.

Effective antiplatelet therapy is crucial following PCI, and aspirin is the most widely utilized first-line antiplatelet agent and serves as the primary choice for antiplatelet therapy post-PCI (Potpara et al., 2020). Ticagrelor is among the latest antiplatelet agents utilized to inhibit platelet aggregation by blocking the ADP receptors of the P2Y12 subtype (Kilic et al., 2018). Tirofiban, a well-established GPIIb/IIIa receptor antagonist, has demonstrated efficacy in enhancing coronary blood flow and myocardial reperfusion during emergency PCI in patients with STEMI (Peng and Li, 2022). Research indicates that tirofiban effectively inhibits the release of serotonin and thromboxane A2 from platelets, thereby mitigating vasoconstriction and facilitating distal coronary vasodilation. Additionally, tirofiban has been shown to improve vascular endothelial function by augmenting endogenous nitric oxide (NO) levels. Empirical evidence indicates that the concomitant use of ticagrelor and tirofiban enhances coronary recanalization rates and mitigates the occurrence of no-reflow or slow blood flow during PCI, without a significant increase in adverse reactions (Li et al., 2022; Peng and Li, 2022). In this particular case, the patient was administered DAPT comprising ticagrelor and aspirin, in addition to intravenous tirofiban post-PCI, thereby demonstrating the safety and efficacy of this antithrombotic strategy.

For patients experiencing AMI, particularly during the perioperative period, the implementation of appropriate antithrombotic therapy is crucial to mitigate the risk of recurrent thrombotic events. Current guidelines recommend administering potent P2Y12 inhibitors, such as ticagrelor or prasugrel, over clopidogrel for AMI patients post-PCI. This preference is due to their superior efficacy in reducing thrombotic risk, particularly during the early stages of acute coronary syndrome (Casula et al., 2024; Morrow et al., 2009; Jobs et al., 2024). Several randomized trials have demonstrated that potent P2Y12 inhibitors are associated with a higher risk of bleeding compared to clopidogrel (James et al., 2011). However, the majority of bleeding events in acute coronary syndrome occur during the maintenance phase, which is more than 1 month after DAPT and PCI (Turgeon et al., 2020; Andell et al., 2015). Consequently, these findings have informed a strategy of tapering the intensity of DAPT over time, wherein potent P2Y12 inhibitors are administered during the acute phase of acute coronary syndrome, and clopidogrel is utilized during the chronic treatment period (Kim et al., 2021). In a multicenter, non-inferiority randomized trial, for patients with stable acute myocardial infarction who did not experience major ischemic or bleeding events within 1 month post-PCI, switching from ticagrelor to clopidogrel could significantly reduce bleeding risk without increasing the incidence of ischemia or recurrent thrombotic events have been demonstrated (Turgeon et al., 2020). In this particular patient, no hemorrhagic complications were observed with DAPT using ticagrelor and aspirin 1 month following urological surgery and PCI. This observation is consistent with previous studies, thereby supporting the safety of perioperative DAPT. Subsequently, ticagrelor was transitioned to clopidogrel, based on evidence indicating that this switch mitigates the risk of bleeding without elevating the incidence of ischemic events (Turgeon et al., 2020). This case illustrates that DAPT with ticagrelor and aspirin constitutes a safe and effective antithrombotic regimen for patients during the perioperative period.

A notable limitation of this study was the patient’s refusal to undergo CAG, despite the presence of multiple coronary luminal stenoses identified on coronary CTA. This refusal may have been a contributing factor to the occurrence of perioperative STEMI on postoperative day 1 (POD1). This study underscores the critical importance of performing CAG in patients with significant coronary artery disease prior to major surgery to optimize perioperative management and mitigate the risk of such complications.

## 4 Conclusion

In this study, PCI in conjunction with DAPT utilizing ticagrelor and aspirin was demonstrated to be a safe and efficacious antithrombotic strategy for postoperative AMI during the perioperative period. Meanwhile, the implementation of a rigorous and standardized laparoscopic radical cystectomy with Bricker conduit urinary diversion provides a structured approach to a challenging scenario. These findings suggest a potential paradigm shift in the management of antithrombotic therapy for high-risk surgical patients. We advocate for considering the continuation of antithrombotic therapy, particularly DAPT, during the perioperative period of major surgical procedures, provided that appropriate precautions are implemented.

## Data Availability

The original contributions presented in the study are included in the article/supplementary material, further inquiries can be directed to the corresponding authors.
